# VERRUCOUS CARCINOMA OF THE ESOPHAGUS INVOLVING THE ENTIRE ESOPHAGUS

**DOI:** 10.1590/S0102-6720201500030019

**Published:** 2015

**Authors:** André BRANDALISE, Cláudia LORENZETTI, Nilton Cesar ARANHA, Nelson Ary BRANDALISE

**Affiliations:** Medical Center Hospital of Campinas, Campinas, SP, Brazil.

## INTRODUCTION

Verrucous carcinoma is a very rare type of squamous cell carcinoma, slow-growing and found in the oropharynx, larynx, penis, scrotum, vulva, vagina, cervix, endometrium, bladder and anorectal region. It is believed to be related to chronic irritation or inflammation of the mucosa due to smoking, drinking, achalasia, esophagitis, ingestion of lye or esophageal diverticulum.

The main symptom is dysphagia, associated with great weight loss. Patients are usually admitted in an advanced state of malnutrition, especially due to delays in diagnosis, leading to high surgical risk and mortality^1^^,^ 2 ^,^ 4 ^,^ 5 ^,^ 6 ^,^ 7 ^,^ 8 ^,^ 9 ^,^ 10

## CASE REPORT

Man aged 64, smoker for 44 years, having stopped for 16 years. He presented with rapidly progressive dysphagia, from solid to liquid in four months, associated with weight loss of 18 kg in the period.

Initially admitted for investigation at another facility where he underwent upper gastrointestinal endoscopy, which showed whitish plaques in the entire esophagus and biopsies showing *Candida sp*. He was treated with intravenous antifungal and remained hospitalized for 28 days, but there was no improvement of the dysphagia, on the contrary, it progressed to inability to swallow saliva. A new endoscopy revealed growth of a vegetative mass in the mucosa and it was not possible to reach the stomach. A nasogastric feeding tube was inserted at this moment. Again biopsies were taken and the results showed no malignancy.

**FIGURE 1:**
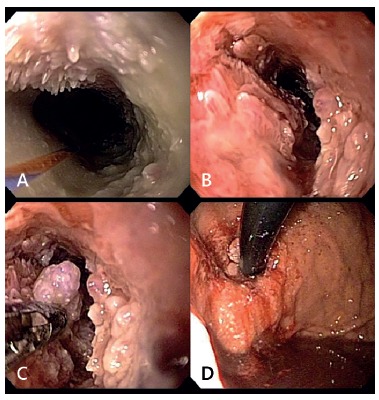
- Endoscopic appearance: a) proximal esophagus; b) middle esophagus; c) distal esophagus; e d) preserving the gastric epithelium in the cardia

Upon arrival in the institution, the patient was quite emaciated and another endoscopy was performed showing whitish papillomatous lesions, starting just below the cricopharyngeal muscle, increasing in size and becoming exophytic, multilobular polypoid mass, narrowing the esophageal lumen (Figure 1). New biopsies revealed papillary squamous epithelium, consistent with esophageal squamous papilloma without atypia.

A tomography was performed, showing extensive esophageal lesion with 15 cm long, from the middle segment, completely obstructing the esophageal lumen. Esophageal walls were also thickened in the upper segment, but with esophageal lumen, preserved 

As the successive biopsies were negative for carcinoma, discussion was held with pathology team, presenting the CT and endoscopic findings. Due to the high degree of suspicion, the pathologist raised the possibility of verrucous carcinoma of the esophagus and the patient was scheduled for an esophagectomy.

The procedure started by videothoracoscopy, but after long and laborious dissection, there was no security in finding the plane between the neoplastic mass and left atrium and pulmonary veins.

A thoracotomy was carried out with full mobilization of the esophagus and lymphadenectomy. The patient was then placed in the supine position and the operation was completed by abdominal laparoscopy and neck incision for reconstruction with isoperistaltic gastric tube. The specimen was removed, protected, through the abdomen. As can be seen in Figure 3, the margin of esophageal section was grossly affected by cancer, and even enlarging the resection, surgical margin persisted macroscopically affected.

**FIGURE 2:**
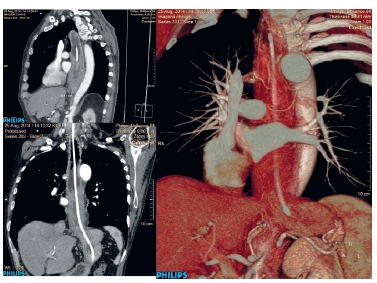
- CT scan appearance

**FIGURE 3:**
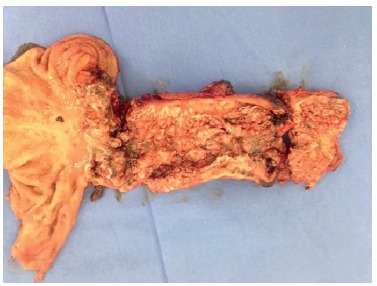
- Surgical specimen

As the patient refused pharyngolaryngectomy, manual anastomosis was performed 1-2 cm below the cricopharyngeal muscle, with the intention to perform ablation of residual mucosa later. The postoperative period was uneventful, without anastomotic fistula or pulmonary complications.

The pathology report showed squamous cell verrucous carcinoma, well differentiated, infiltrating the adventitia of the esophagus, with 16 cm long. There was no invasion of the gastric epithelium at the cardia. The proximal margin presented the same pattern of papillary cell cancer, but restricted mucosa. It was not detected angiolymphatic nor perineural invasion, pT3pN0 stage.

In the postoperative follow-up, two sessions of endoscopic ablation with argon plasma were performed, with good results at the time of ablation, but recurrence of papilloma at control exams.

After these attempts the patient was sent to adjuvant radiotherapy. After completion of radiation therapy, control endoscopy showed complete disappearance of papilloma in the mucosa.

**FIGURE 4:**
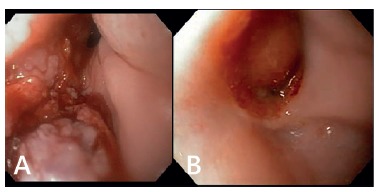
- Esophagogastric anastomosis: a) residual papilomathoid lesions; b) after radiotherapy

The patient is doing well, with no signs of disease on 12 months follow-up (Figure 4). 

## DISCUSSION

Verrucous carcinoma is very rare type of squamous cell carcinoma. It was first described by Minilley et al. in 1967^1^. Of unknown etiology may be related to smoking, alcohol consumption, achalasia, esophagitis, lye ingestion or esophageal diverticulum^2,^ 3 ^,^ 4 ^,^ 6 ^,^ 7 ^,^ 9.

There are fewer than 30 cases reported in the literature and there is a strong link with smoking and alcohol consumption. HPV can also have influence on the development and progression to cancer^2^^,^ 9 ^,^ 10. This patient had been a smoker for 44 years, but despite pappiltomatous lesions, HPV was not observed.

The main form of presentation is dysphagia associated with major weight loss^10^. Patients are usually admitted in an advanced state of malnutrition, especially due to delays in diagnosis, leading to the high surgical mortality risk^9^. 

Endoscopically, the appearance is characteristic with large exophytic masses, not ulcerated, of verruciform multilobulated. In this patient injury assailed the entire esophagus and respected precisely the epithelium of cardia.

A recent study of 11 cases from a single institution treated in 15-year period brings together the main characteristics of this type of carcinoma. Commonly (73%) associated with infection by *Candida sp,* which sometimes delays the final diagnosis. In five of the 11 patients had affected the entire length of the esophagus^10^. Only six of the 11 patients were treated by surgery.

The conclusive preoperative diagnosis of verrucous carcinoma is hard to do. Superficial biopsies show only nonspecific acanthosis, hyperkeratosis or parakeratosis associated with acute or chronic inflammation. The diagnosis should be made based on the characteristics evaluated all together. Endoscopic appearance and image, which can be tomography or endoscopic ultrasound, reveals transmural involvement of the lesion. Deeper biopsies by aspiration can be attempted with endoscopic ultrasound. Another method that can provide greater amount of tissue for histological evaluation is endoscopic mucosal resection.

In this case the diagnosis and therapy were challenging, since endoscopic biopsies were not conclusive and the final diagnostic came only after analyzing the resected specimen. Esophagectomy can be curative, but has high mortality rates and, in this case, the extremely infiltrative nature of the hardened mass made the surgical removal more difficult. 

A peculiarity of this case, not reported in the literature yet, was the response to adjuvant therapy with disappearance of lesions in the residual esophageal mucosa, which can be the basis for neoadjuvant therapy in order to allow more conservative resections when there is involvement of the entire esophagus. There is already reported in the literature neoadjuvant treatment, with good response^8^, but there is no consensus in the literature in this regard.

## References

[1] Agha FP, Weatherbee L, Sams JS (1984). Verrucous carcinoma of the esophagus. Am J Gastroenterol.

[2] Ahmed K, Timmerman G, Meyer R, Miller T, Mazurczak M, Tams K (2013). Verrucous carcinoma of the esophagus: a potential diagnostic
dilemma. Case reports in gastroenterology.

[3] Behrens A (2013). Endoscopic Imaging of Esophageal Verrucous Carcinoma. Video Journal and Encyclopedia of GI Endoscopy.

[4] Chu Q, Jaganmohan S, Kelly B, Hobley J (2011). Verrucous carcinoma of the esophagus: a rare variant of squamous cell
carcinoma for which a preoperative diagnosis can be a difficult one to
make. The Journal of the Louisiana State Medical Society : official organ of the
Louisiana State Medical Society.

[5] Devlin S, Falck V, Urbanski SJ, Mitchell P, Romagnuolo J (2004). Verrucous carcinoma of the esophagus eluding multiple sets of
endoscopic biopsies and endoscopic ultrasound: a case report and review of the
literature. Canadian journal of gastroenterology = Journal canadien de
gastroenterologie.

[6] Lagos AC, Marques IN, Reis JD, Neves BC (2012). Verrucous carcinoma of the esophagus. Rev Esp Enferm Dig.

[7] Macias-Garcia F, Martinez-Lesquereux L, Fernandez B, Parada P, Larino-Noia J, Sobrino-Faya M (2010). Verrucous carcinoma of the esophagus: a complex
diagnosis. Endoscopy.

[8] Minielly JA, Harrison EG Jr, Fontana RS, Payne WS (1967). Verrucous squamous cell carcinoma of the esophagus. Cancer.

[9] Ramani C, Shah N, Nathan RS (2014). Verrucous carcinoma of the esophagus: A case report and literature
review. World journal of clinical cases.

[10] Sweetser S, Jacobs NL, Wong Kee Song LM (2014). Endoscopic diagnosis and treatment of esophageal verrucous squamous
cell cancer. Dis Esophagus.

[11] Tonna J, Palefsky JM, Rabban J, Campos GM, Theodore P, Ladabaum U (2010). Esophageal verrucous carcinoma arising from hyperkeratotic plaques
associated with human papilloma virus type 51. Dis Esophagus.

